# A service evaluation and improvement project: outcomes of older adult functional referrals to the North Derbyshire Liaison Team

**DOI:** 10.1192/bjo.2021.843

**Published:** 2021-06-18

**Authors:** Maha Ejaz, Joseph Atkins, Bushra Azam, Stuart Laverack

**Affiliations:** 1 University of Sheffield; 2 Derbyshire Healthcare NHS Foundation Trust

## Abstract

**Aims:**

This project aims to examine a group of service users over the age of 65 with functional presentations, who were assessed by the Liaison Psychiatry team between June 2018 and 2019.

Hypotheis: We believe that there is a need for a community crisis service for the older adult North Derbyshire population with functional presentations.

**Background:**

Due to the lack of community crisis services for patients over 70, it was felt that a significant number of these patients were admitted to inpatient psychiatric units from medical wards who would benefit from crisis intervention instead. We wanted to see the clinical outcomes of this population, referred to the liaison team, determining whether this was significant concern. If this need is established, based on the data collected, this will enable the trust to look into starting a service for this age group to provide care in their own home. In turn, it will help to reduce unnecessary admissions to acute mental health wards and reduce stays in the general hospital – preventing consequences associated with long term hospital stays.

**Method:**

Retrospective analysis using PARIS notes of 366 patients referred to the liaison team were scrutinised to determine the assessment diagnosis and outcome of patients with functional conditions. The inclusion criteria were patients over the age of 65 referred with functional psychiatric illnesses between June 2018 and 2019. We excluded 84 patients assessed to have delirium or organic presentations from our analysis. Data were collected and analysed using Excel.

**Result:**

Among the referrals to the liaison team, the majority of patients were referred with mood disorders followed by self-harm, psychosis and anxiety. Although the majority of patients were referred back to either the community mental health team or primary care, 11% of the sample were admitted to inpatient psychiatric units. This number may have been lower and admission may have been avoided if a community crisis service was in place for this population.

**Conclusion:**

In conclusion, the data support our initial concerns that there is a need for crisis services for this age group with functional presentations. There is ongoing discussions around a need to develop this service and therefore our results will contribute to the development of an older adult functional service in Derbyshire.

